# Impact of reducing day 1 dexamethasone dose in anthracycline-containing regimens on acute gastrointestinal symptoms associated with breast cancer treatment

**DOI:** 10.1038/s41598-021-02765-3

**Published:** 2021-12-02

**Authors:** Yoshitaka Saito, Yoh Takekuma, Takashi Takeshita, Mitsuru Sugawara

**Affiliations:** 1 Department of Pharmacy, Hokkaido University Hospital, Kita 14-jo, Nishi 5-chome, Kita-ku, Sapporo, 060-8648 Japan; 2 Department of Breast Surgery, Hokkaido University Hospital, Kita 14-jo, Nishi 5-chome, Kita-ku, Sapporo, 060-8648 Japan; 3 Laboratory of Pharmacokinetics, Faculty of Pharmaceutical Sciences, Hokkaido University, Kita 12-jo, Nishi 6-chome, Kita-ku, Sapporo, 060-0812 Japan

**Keywords:** Cancer, Medical research, Oncology

## Abstract

The potential of steroid sparing from day 2 onward is reported in anthracycline-containing regimens for breast cancer treatment. We evaluated whether the reduction of dexamethasone (DEX) dose from 9.9 to 6.6 mg on day 1 is possible in anthracycline-containing treatments. Patients receiving anthracycline-containing regimens were divided into control (9.9 mg DEX on day 1) and reduced (6.6 mg DEX on day 1) groups, and retrospectively evaluated. The complete response (CR) rate and the incidence and severity of nausea, vomiting, anorexia, and fatigue were evaluated. The CR rate in the acute phase (day 1) was 63.1% and 38.1% in the control and reduced groups, respectively, with significant difference (*P* = 0.01) between the groups. However, no difference was found in the delayed phase (days 2–7). The incidence of anorexia and vomiting during treatment was not statistically different. Severity of nausea tended to, but not statistically, worsen while anorexia significantly worsened in the reduced group. Multivariate analysis suggested that patients < 55 years, with non- or less-alcohol drinking habit (< 5 days/week), and administered reduced-DEX dosage on day 1, have a higher risk of acute nausea development. Thus, reducing day 1 DEX dose in anthracycline-containing regimens is not suitable for acute nausea management.

## Introduction

Chemotherapy-induced nausea and vomiting (CINV) is one of the most troublesome adverse effects of cancer therapy. Administration of serotonin (5-hydroxytryptamine; 5HT_3_) receptor antagonists (5HT_3_RA), dexamethasone (DEX), and aprepitant (neurokinin-1 receptor antagonist) is one of the most effective prophylactic antiemetic regimens recommended in current guidelines for high emetogenic risk (HEC) chemotherapy^1–4^. The combination of anthracyclines and cyclophosphamide, which are key regimens in breast cancer treatment^5–8^, is categorized as an HEC regimen. Patient characteristics including younger age and female sex have been suggested to be risk factors for CINV^9,10^. However, patients with a drinking habit experience less cisplatin (CDDP)-induced CINV compared to those without this habit^9^. Breast cancer is the most common cancer among women and one of the most common causes of death among them^11^. The incidence rate of breast cancer increases with age and reaches its peak around the age of menopause and then gradually decreases or remains constant. Consequently, many breast cancer patients are at risk for CINV in their chemotherapeutic treatment. Management of CINV is one of the most important mission as medication treatment is carried out for outpatients in most cases.

Palonosetron has been reported to be superior to granisetron, which is classified as a first generation 5-HT_3_RA, in combination with aprepitant and DEX in the HEC regimen^12^. However, steroid sparing, which reduces DEX dose duration, is also suggested to be possible in anthracycline- and CDDP-containing regimens^13–15^. At the Hokkaido University Hospital, antiemetic treatment for anthracycline-containing regimens includes palonosetron, DEX, and aprepitant; however, previously, the DEX dosage was 6.6 mg infusion on day 1 and 4 mg orally on days 2–4. This DEX dosage has been changed to 9.9 mg infusion on day 1 and 8 mg orally on days 2–4 in accordance with the guidelines^4^. From the studies described above, dose escalation on days 2–4 is speculated to be ineffective. However, a suitable DEX dosage for day 1 is still unclear. In this study, we evaluated whether DEX dose reduction on day 1 can be performed in anthracycline-containing regimens.

## Results

### Patient characteristics

**Figure 1:**
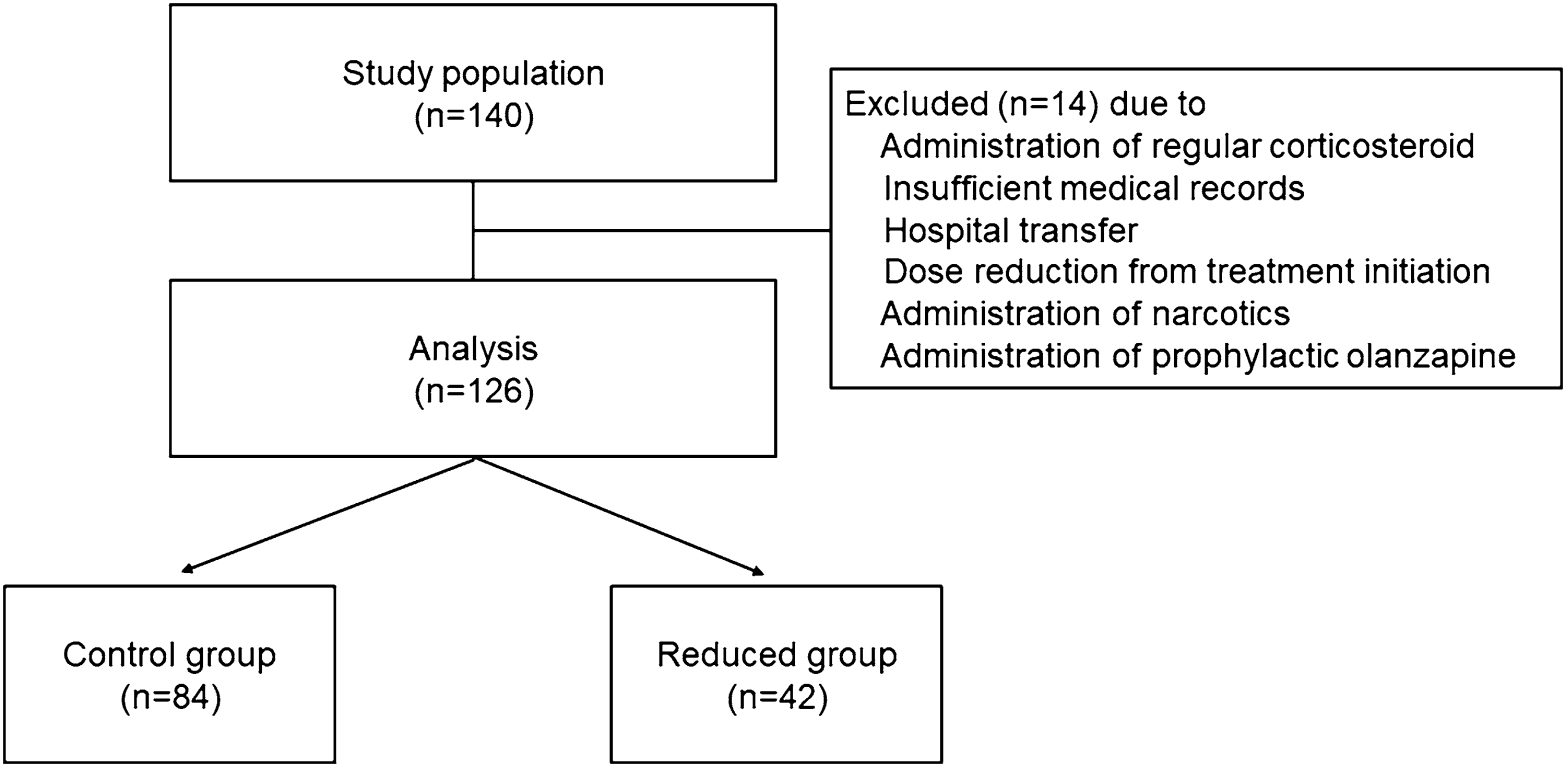
Design of this study.

One hundred and twenty-six of the 140 patients were enrolled according to the eligibility criteria of this study (Fig. 1). The baseline patient characteristics are shown in Table 1. There were no significant differences between the two groups in Eastern Cooperative Oncology Group performance status (ECOG PS), staging, presence of lymph node metastases, treatment setting, hormonal receptor expression, human epidermal growth factor receptor 2 (HER2) overexpression, prior treatment, menopause, birth history, body surface area (BSA), liver dysfunction (grade 1 or higher aspartate aminotransferase, alanine aminotransferase, γ-glutamyltransferase, total bilirubin elevation), renal dysfunction (grade 1 or higher serum creatinine elevation), serum albumin, regular alcohol intake (≥ 5 days in a week), smoking history, and regular administration of antacids. Patients in the control group were significantly older; however, patients < 55 years old, which is suggested to be a risk for acute nausea^16^, did not differ between the groups. Patients in the reduced group received more FEC regimen (epirubicin (100 mg/m^2^) + cyclophosphamide (500 mg/m^2^) + 5-fluorouracil (500 mg/m^2^), every 3 weeks) than EC (epirubicin (90 mg/m^2^) + cyclophosphamide (600 mg/m^2^), every 3 weeks) or dose-dense AC (doxorubicin (60 mg/m^2^) + cyclophosphamide (600 mg/m^2^, every 2 weeks) regimens, and had greater HER2 overexpression.

**Table 1:** Patient characteristics.

	Control group (n = 84)	Reduced group (n = 42)	*P*-value
Age (median, range)	54 (26–73)	51 (32–66)	0.04*
Patients < 55 years old	42	24	0.57
**Performance status**			
0–1	84	42	1.00
**Staging**			
I–III	79	39	
IV/Recurrence	5	3	1.00
Presence of Lymph node metastases	**43**	**19**	**0.57**
**Treatment setting**			
Adjuvant/ Neo-adjuvant	79	39	
Metastatic/Recurrence	5	3	1.00
**Hormonal receptors**			
ER, PR-positive or both	45	17	0.19
HER2 overexpression	19	18	0.02*
Prior treatment existence	9	3	0.75
Menopause	52	22	0.34
Birth history	51	31	0.17
BSA (m^2^) (median, range)	1.54 (1.33–2.02)	1.55 (1.34–1.92)	0.61
Liver dysfunction	30	16	0.85
Renal dysfunction	10	2	0.33
Serum albumin (g/dL) (median, range)	4.2 (3.5–4.8)	4.2 (3.8–4.9)	0.35
Alcohol intake (≥ 5 days in a week)	17	9	1.00
Smoking history (former or current)	43	20	0.85
Regular antacid administration	3	0	0.55
**Treatment regimen**			
AC or EC	70	6	
FEC	14	36	< 0.01**

*P < 0.05.

**P < 0.01.

Significant values are in bold.

*ER* estrogen receptor, *PR* progesterone receptor, *HER2* human epidermal growth factor receptor 2, *BSA* body surface area.

Liver dysfunction: grade 1 or higher aspartate aminotransferase, alanine aminotransferase, γ-glutamyltransferase, total bilirubin elevation.

Renal dysfunction: grade 1 or higher serum creatinine elevation.

Antacids include proton pump inhibitors and histamine type 2 receptor antagonists.

### Comparison of the CINV incidence

**Figure 2:**
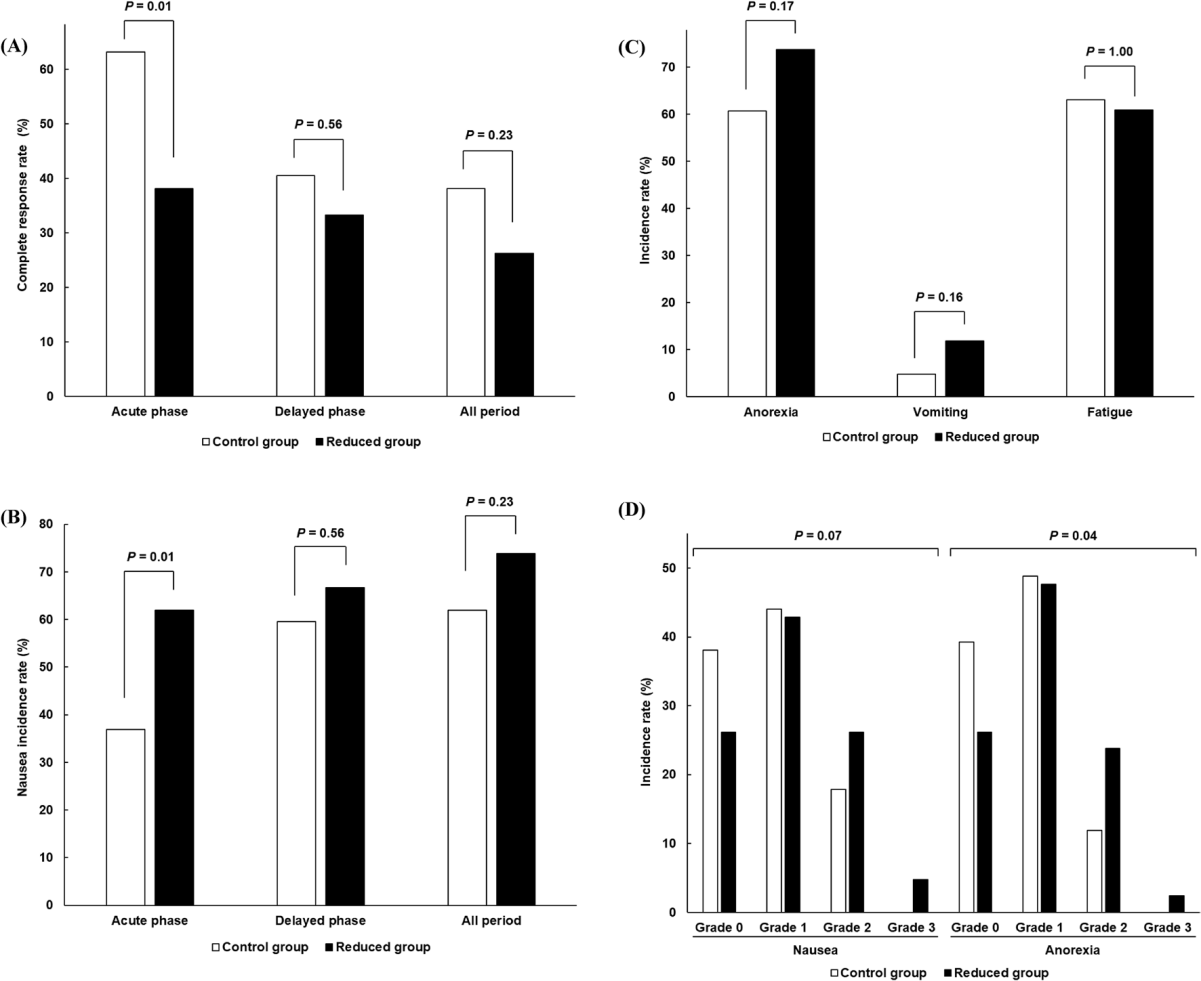
Comparison of the (**A**) CR rate; the incidence of (**B**) nausea, (**C**) anorexia, vomiting, and fatigue; and the (**D**) severity of nausea and anorexia.

Figure 2 shows the comparison of digestive symptoms and fatigue incidence and severity between the two groups. Difference in the rate of complete response (CR), which was defined as the absence of emetic events, vomiting, and need for rescue antiemetic treatment, on day 1 (acute phase) between the two groups was defined as the primary endpoint of this study: the rate was 63.1% in the control group, and 38.1% in the reduced group, which was significantly lowered by DEX dose reduction to 6.6 mg (*P* < 0.01); the rate in the delayed phase (within days 2–7) and all evaluation periods was not different (Fig. 2A). The incidence of nausea in the acute and delayed phases and all evaluation periods was 36.9%, 59.5%, and 61.9% in the control group, and 61.9%, 66.7%, and 73.8% in the reduced group, respectively, suggesting that acute nausea more significantly appeared in the reduced group (*P* < 0.01, Fig. 2B). With regard to anorexia and vomiting, the incidence in all evaluation periods was 60.7% and 4.8% in the control group and 73.8% and 11.9% in the reduced group, which was not statistically different (Fig. 2C). Fatigue incidence was also similar between the groups. The severity of nausea tended to, but not statistically, worsen, whereas anorexia significantly became more severe in the reduced group (Fig. 2D).

### Assessment of the risk factors for nausea incidence

Multivariate analysis was performed to identify independent risk factors for nausea incidence in the acute phase and all evaluation periods. Patients younger than 55 years, with non- or less-alcohol drinking habit (less than 5 days within a week), and administered reduced-DEX dose to 6.6 mg on day 1, were identified to be at higher risk for acute nausea development (Table 2). With regard to factors in all evaluation periods, patients younger than 55 years old were revealed to be at risk (Table 3).

**Table 2:** Univariate and multivariate analyses of the risk factors associated with the frequency of nausea in the acute phase.

(A)	Acute nausea incidence (n, %)	Univariate analysis		Multivariate analysis	
		Odds ratio (95% CI)	*P*-value	Odds ratio (95% CI)	*P*-value
**Age (years)**					
< 55	38 (57.6%)				
≥ 55	20 (33.3%)	2.71 (1.31–5.61)	0.001**	3.12 (1.42–6.86)	0.005**
**Treatment setting**					
Metastatic/recurrence	3 (37.5%)				
Adjuvant/neoadjuvant	55 (46.6%)	0.69 (0.16–3.01)	0.62	Excluded	–
**Prior treatment**					
Present	5 (41.7%)				
Absent	53 (46.5%)	0.82 (0.25–2.74)	0.75	Excluded	–
**Hormonal receptors**					
ER, PR-positive or both	31 (50.0%)				
Negative	27 (42.2%)	1.37 (0.68–2.77)	0.38	Excluded	–
**HER2 overexpression**					
Positive	21 (56.8%)				
Negative	37 (41.6%)	1.84 (0.85–4.00)	0.12	1.46 (0.61–3.47)	0.40
**BSA (m**^**2**^**)**					
> 1.5	33 (40.7%)				
≤ 1.5	25 (55.6%)	0.55 (0.26–1.15)	0.11	0.45 (0.20–1.04)	0.06
**Alcohol intake (≥ 5 days in a week)**					
Absent	50 (50.0%)				
Present	8 (30.8%)	2.25 (0.90–5.65)	0.08	2.97 (1.09–8.13)	0.03*
**Smoking history**					
Current or former	26 (41.3%)				
Never	32 (50.8%)	0.68 (0.34–1.38)	0.28	Excluded	–
**Birth history**					
Present	38 (46.3%)				
Absent	20 (45.5%)	1.04 (0.50–2.16)	0.92	Excluded	–
**Liver dysfunction**					
Present	19 (41.3%)				
Absent	39 (48.8%)	0.74 (0.36–1.54)	0.42	Excluded	–
**Regular administration of antacids**					
Present	2 (66.7%)				
Absent	56 (45.5%)	2.39 (0.21–27.08)	0.48	Excluded	–
**Dexamethasone dosage on day 1**					
6.6 mg	26 (61.9%)				
9.9 mg	32 (38.1%)	2.64 (1.23–5.66)	0.01*	2.74 (1.17–6.43)	0.02*

*P < 0.05, **P < 0.01.

*CI* confidential interval, *ER* estrogen receptor, *PR* progesterone receptor, *HER2* human epidermal growth factor receptor 2, *BSA* body surface area.

Liver dysfunction: grade 1 or higher aspartate aminotransferase, alanine aminotransferase, γ-glutamyltransferase, total bilirubin elevation.

Antacids include proton pump inhibitors and histamine type 2 receptor antagonists.

**Table 3:** Univariate and multivariate analyses of the risk factors associated with the frequency of nausea in all evaluation periods.

(B)	Nausea incidence (n, %)	Univariate analysis		Multivariate analysis	
		Odds ratio (95% CI)	*P*-value	Odds ratio (95% CI)	*P*-value
**Age (years)**					
< 55	52 (78.8%)				
≥ 55	31 (51.7%)	3.48 (1.60–7.56)	0.001**	2.95 (1.31–6.64)	0.009**
**Treatment setting**					
Metastatic/recurrence	5 (62.5%)				
Adjuvant/neoadjuvant	78 (66.1%)	0.85 (0.19–3.76)	0.84	Excluded	–
**Prior treatment**					
Present	7 (58.3%)				
Absent	76 (66.7%)	0.79 (0.21–2.35)	0.56	Excluded	–
**Hormonal receptors**					
ER, PR-positive or both	46 (74.2%)				
Negative	37 (57.8%)	2.10 (0.99–4.46)	0.05	1.71 (0.75–3.90)	0.20
**HER2 overexpression**					
Positive	24 (64.9%)				
Negative	59 (66.3%)	0.94 (0.42–2.10)	0.88	Excluded	–
**BSA (m**^**2**^**)**					
> 1.5	51 (63.0%)				
≤ 1.5	32 (71.1%)	0.69 (0.31–1.52)	0.36	Excluded	–
**Alcohol intake (≥ 5 days in a week)**					
Absent	67 (67.0%)				
Present	16 (61.5%)	1.27 (0.52–3.10)	0.60	Excluded	–
**Smoking history**					
Current or former	42 (66.7%)				
Never	41 (65.1%)	1.07 (0.51–2.24)	0.85	Excluded	–
**Birth history**					
Present	56 (68.3%)				
Absent	27 (61.4%)	1.36 (0.63–2.91)	0.43	Excluded	–
**Liver dysfunction**					
Present	29 (63.0%)				
Absent	54 (67.5%)	0.82 (0.38–1.76)	0.61	Excluded	–
**Regular administration of antacids**					
Present	2 (66.7%)				
Absent	81 (65.9%)	1.04 (0.09–11.77)	0.98	Excluded	-
**Dexamethasone dosage**					
6.6 mg on day 1; 4 mg on days 2–4	31 (73.8%)				
9.9 mg on day 1; 8 mg on days 2–4	52 (61.9%)	1.73 (0.77–3.92)	0.19	1.83 (0.77–4.36)	0.17

*P < 0.05, **P < 0.01.

*CI* confidential interval, *ER* estrogen receptor, *PR* progesterone receptor, *HER2* human epidermal growth factor receptor 2, *BSA* body surface area.

Liver dysfunction: grade 1 or higher aspartate aminotransferase, alanine aminotransferase, γ-glutamyltransferase, total bilirubin elevation.

Antacids include proton pump inhibitors and histamine type 2 receptor antagonists.

## Discussion

It is necessary to manage CINV to deliver safer and less onerous anticancer treatment, especially in outpatient chemotherapy. Advances in antiemetic therapy have significantly improved the quality of life of patients during treatment. However, its administration induces other adverse effects and drug-drug interactions. In particular, corticosteroid administration induces blood sugar elevation, insomnia, and increased susceptibility to infection, especially to pneumocystis pneumonia (PCP)^17,18^. Owing to these problems, we evaluated whether DEX dose reduction on day 1 is possible for CINV management, especially in the acute phase.

DEX dose reduction on day 1 from 9.9 to 6.6 mg was found to significantly decrease CR rate and increase nausea incidence in the acute phase, which met the primary endpoint of this study. However, the CR rate and nausea incidence in the delayed phase were not found to be statistically different, which is consistent with the findings of previous reports^13–15^. The incidence of anorexia, vomiting, and fatigue in all evaluation periods also did not differ between the groups. With regard to severity, patients in the reduced group developed significantly more severe anorexia, and tended to, but not statistically, experience more severe nausea. Acute nausea has been reported to affect subsequent delayed nausea^19^; thus, patients in the reduced group might have developed more severe symptoms due to acute nausea, although it did not affect the incidence. The findings of this study suggest that DEX dose reduction on day 1 is not suitable for acute CINV management.

Treatment and patient factors affect the emetogenic risks of CINV^4^. Treatment factors include emetogenicity and dosages of chemotherapeutic agents, tissue target, and radiation therapy volume. Patient factors, such as age, gender, drinking habit, and experience of nausea gravidarum, influence the incidence of CINV^4,9,10^. Moreover, NCCN guidelines show that bowel obstruction, vestibulopathy, brain metastasis, electrolyte abnormality, uremia, opioid use, gastric atony, and mental disorders are potential risk factors for emesis^3^. Younger age has been reported to be an independent risk factor of CINV^3,10,16,20–22^. In particular, the cutoff age for acute CINV is 50–55 years^3,16^. Sekine et al. reported that patients < 55 years old develop more acute CINV (odds ratio, 95% confidence interval: 2.56, 1.94–3.37), and those with non-habitual alcohol intake also have acute CINV risk (1.90, 1.43–2.51)^16^. Recent NCCN guidelines also indicate that CINV occurrence increases in younger women with a history of no or low alcohol use, motion sickness, or morning sickness^3^. Thus, the results obtained in this study are consistent with prior findings. We also evaluated whether DEX dosage affects the acute CR rate in patients with risk factors, and found that the CR rate in acute phase was significantly decreased by DEX dose reduction on day 1 (52.4% vs 25.0%, *P* = 0.04 in patients aged < 55 years; 59.7% vs 33.3%, *P* = 0.02 in patients with non- or less-alcohol drinking habit), suggesting that DEX dosage on day 1 should not be reduced (Supplemental Table 1). By contrast, CR rates in all evaluation periods in patients aged < 55 years were similar between control and reduced DEX patients. These results suggest that outcomes obtained for all patients correspond with outcomes for patients with risk factors.

On the other hand, breast cancer patients are considered to be at a greater risk for CINV development, and patients treated with AC experienced more acute CINV than those administered CDDP^13^. Therefore, it is unclear whether the results obtained in this study are suitable for CDDP-containing regimens.

Palonosetron has been reported to be superior to granisetron in combination with aprepitant and DEX in the HEC regimen, especially during the delayed phase^12^. The possibility of DEX dose reduction or sparing with first generation 5-HT_3_RA is unclear, but can worsen CINV. Therefore, the sparing should be considered with palonosetron. On the other hand, olanzapine has been suggested to be effective for CINV prevention in HEC regimens although no patients received its prophylactic administration in this study^23,24^. Olanzapine may reduce the incidence of acute nausea regardless of DEX dose reduction on day 1. Further studies are needed to elucidate the best strategy for CINV management.

This study had some limitations regarding the evaluation of the impact of DEX dosage on day 1 on the acute antiemetic effect in anthracycline-containing treatment. First, this study was retrospectively performed. Second, we adopted a physician-based or pharmacist-based evaluation by referring to a treatment diary, which almost all patients wrote, although some of them listed the incidence but not the severity. Therefore, we evaluated the severity according to the patients’ complaint. As symptom evaluation by a medical personnel differs from that by patients suffering from CINV^10,25^, the severity may not have been correctly assessed. Thus, it is necessary to conduct a large-scale, randomized, prospective, multicenter study with a subjective severity assessment by patients. Third, all patients in this study were not administered prophylactic olanzapine; instead, they took metoclopramide. Although the prophylactic antiemetic effect of metoclopramide in HEC regimens is unclear, its administration may have affected the results. Further studies without metoclopramide could provide more appropriate results. Fourth, evaluation in delayed phase is desirable in comparing patients with and without DEX administration. Finally, we could not evaluate the patients’ history of motion sickness and morning sickness with pregnancy, which may have affected the results. In addition, treatment regimens were significantly different between the groups; as both AC/EC and FEC are classified as HEC regimens in the guidelines^1–3^, we consider that the impact on the results is low. Evaluation with well-balanced patients and enough information on the risk factors will enable better outcomes to be derived.

In conclusion, our study suggests that DEX dose reduction on day 1 in anthracycline-containing regimens is not suitable for acute CINV management. A further evaluation of the antiemetic regimens will provide less onerous chemotherapy, especially in outpatient chemotherapy.

## Methods

### Patients

The medical records of 126 patients with breast cancer who received anthracycline-containing regimens were evaluated in this retrospective study. The regimens included EC, FEC, and dose-dense AC. All patients met the following baseline criteria: (1) age ≥ 20 years; (2) 0 to 2 ECOG PS; (3) sufficient renal and liver function. Patients who were previously administered anthracyclines, regularly dosed corticosteroids, antiemetics, and narcotics, transferred to another hospital during the first chemotherapy cycle, those with nausea at baseline, and without sufficient information were excluded. Patients who were administered olanzapine as prophylactic antiemesis were also omitted.

The patients were divided into two groups: reduced, patients who were administered DEX infusion 6.6 mg on day 1 and 4 mg orally on days 2–4 between April 2016 and January 2018; and controls, who were administered DEX infusion 9.9 mg on 1 and 8 mg orally on days 2–4 between July 2017 and March 2021.

The present study was approved by the Institutional Review Board of the Hokkaido University Hospital (approval number: 021-0020), and was carried out in accordance with the Declaration of Helsinki and STROBE statement. In view of the retrospective nature of the study, informed consent from the subjects was waived by the committee.

### Treatment methods

All regimens included palonosetron 0.75 mg on day 1 and aprepitant 125 mg on day 1 and 80 mg on days 2 and 3. DEX was administered as described above. Moreover, metoclopramide 5 mg three times per day from the evening of day 1 to day 8 was administered to all patients. Additional metoclopramide 5 mg, prochlorperazine 5 mg, domperidone 10 mg, and olanzapine 2.5–5 mg were administered as rescue doses depending on the physician’s discretion.

### Evaluation of CINV and other adverse effects

All required information was obtained from the medical records of patients. We recommended that all patients maintain a daily diary provided by NIPPON KAYAKU (Tokyo, Japan). We evaluated adverse effects by referring to the diary and patient’s complaint. Toxicities in the first cycle were assessed in accordance with the Common Terminology Criteria for Adverse Events, version 5.0 by physicians or pharmacists.

In the present study, the primary endpoint was CR rate evaluation in the acute phase between the reduced group and control group. Secondary endpoints included the evaluation of CR rate within the delayed phase and all evaluation periods, and the incidence and severity of nausea, vomiting, anorexia and fatigue between the groups.

### Statistical analysis

We hypothesized that the CR rate during the acute phase would be 60% in the control group and 35–40% in the reduced group, with a patient ratio of 2:1. To achieve 80% power with an alpha error of 5%, the required sample size was 74–104 subjects in the control group and 37–52 subjects in the reduced group. Eighty-four patients in the control group and 42 patients in the reduced group were analyzed.

The differences in baseline patient clinical characteristics between the reduced and control groups were assessed using Fisher’s exact probability test for categorical outcome variables and the Mann–Whitney *U* test for continuous parameters. The CR rate was compared using Fisher’s exact probability test. Assessment of the adverse effects was conducted using Fisher’s exact probability test for the incidence, and the Mann–Whitney *U* test for severity. Univariate and multivariate logistic analyses were carried out to derive the independent risk factor(s). Potential baseline risk factors included age, treatment setting, prior treatment existence, hormonal receptor expression, HER2 overexpression, BSA, regular alcohol intake, smoking history, birth history, liver dysfunction, regular administration of antacids such as proton pump inhibitors or histamine type 2 receptor antagonists, and DEX dosage according to previous reports^3,4,16^. Variables that had potential associations with nausea incidence in the acute phase and all evaluation periods, as suggested by univariate logistic regression analysis (*P* < 0.20), were considered when building the multivariable model. All analyses were carried out using JMP version 14.0 statistical software (SAS Institute Japan, Tokyo, Japan). Differences were considered to be statistically significant when the *P*-value was less than 0.05.

### Ethics approval and consent to participate

All procedures performed in this study were carried out in accordance with the ethical standards of the institutional and/or national research committee and the 1964 Helsinki declaration and its later amendments or comparable ethical standards. The study was approved by the Institutional Review Board of the Hokkaido University Hospital (approved number: 021-0020). For this type of study, formal consent was waived by the committee.

## Supplementary Information

Supplementary Information.

## Data Availability

The datasets used and/or analyzed in the current study are available from the corresponding author on reasonable request.

## Abbreviations

CINV: Chemotherapy-induced nausea and vomiting
5HT_3_: Serotonin (5-hydroxytryptamine)
5HT_3_RA: 5HT_3_ receptor antagonists
DEX: Dexamethasone
HEC: High emetogenic risk
CDDP: Cisplatin
ECOG PS: Eastern Cooperative Oncology Group performance status
HER2: Human epidermal growth factor receptor 2
BSA: Body surface area
FEC: Epirubicin (100 mg/m^2^) + cyclophosphamide (500 mg/m^2^) + 5-fluorouracil (500 mg/m^2^)
EC: Epirubicin (90 mg/m^2^) + cyclophosphamide (600 mg/m^2^)
AC: Doxorubicin (60 mg/m^2^) + cyclophosphamide (600 mg/m^2^)
CR: Complete response
PCP: Pneumocystis pneumonia
