# Supplementary material for: Impact of reducing day 1 dexamethasone dose in anthracycline-containing regimens on acute gastrointestinal symptoms associated with breast cancer treatment
Source: Sci Rep. 2021 Dec 2;11:23298. doi: 10.1038/s41598-021-02765-3 (PMC8640031; doi:10.1038/s41598-021-02765-3)
Supplement: Supplementary file 1 — Supplementary Information. [file 41598_2021_2765_MOESM1_ESM.docx]

**Supplemental Table 1 Complete response in patients with age <55 years old and non- or less-alcohol drinking habit**

|  | Complete response (n, %) | | |
| --- | --- | --- | --- |
|  | Control patients | Reduced DEX patients | *P*-value |
| Patients aged <55 years (n=66)  Acute phase  All evaluation periods | 22 (52.4)  9 (21.4) | 6 (25.0)  5 (20.8) | 0.04*  1.00 |
| Patients with non- or less-alcohol drinking habit (n=100)  Acute phase | 40 (59.7) | 11 (33.3) | 0.02* |

*: *P*<0.05
